# A Survey on Model Based Approaches for 2D and 3D Visual Human Pose Recovery

**DOI:** 10.3390/s140304189

**Published:** 2014-03-03

**Authors:** Xavier Perez-Sala, Sergio Escalera, Cecilio Angulo, Jordi Gonzàlez

**Affiliations:** 1 Fundació Privada Sant Antoni Abat, Vilanova i la Geltrú, Universitat Politècnica de Catalunya, Vilanova i la Geltrú 08800, Catalonia, Spain; 2 Department Mathematics (MAIA), Universitat de Barcelona and Computer Vision Center (CVC), Barcelona 08007, Catalonia, Spain; E-Mail: sergio@maia.ub.es; 3 Automatic Control Department (ESAII), Universitat Politècnica de Catalunya, Vilanova i la Geltrú 08800, Catalonia, Spain; E-Mail: cecilio.angulo@upc.edu; 4 Department Computer Science, Universitat Autònoma de Barcelona and Computer Vision Center (CVC), Bellaterra 08193, Catalonia, Spain; E-Mail: Jordi.Gonzalez@uab.cat

**Keywords:** human pose recovery, human body modelling, behavior analysis, computer vision

## Abstract

Human Pose Recovery has been studied in the field of Computer Vision for the last 40 years. Several approaches have been reported, and significant improvements have been obtained in both data representation and model design. However, the problem of Human Pose Recovery in uncontrolled environments is far from being solved. In this paper, we define a general taxonomy to group model based approaches for Human Pose Recovery, which is composed of five main modules: appearance, viewpoint, spatial relations, temporal consistence, and behavior. Subsequently, a methodological comparison is performed following the proposed taxonomy, evaluating current SoA approaches in the aforementioned five group categories. As a result of this comparison, we discuss the main advantages and drawbacks of the reviewed literature.

## 1. Introduction

Human pose recovery, or pose recovery in short, refers to the process of estimating the underlying kinematic structure of a person from a sensor input [1]. Vision-based approaches are often used to provide such a solution, using cameras as sensors [2]. Pose recovery is an important issue for many computer vision applications, such as video indexing [3], surveillance [4], automotive safety [5] and behavior analysis [6], as well as many other Human Computer Interaction applications [7,8].

Body pose estimation is a challenging problem because of the many degrees of freedom to be estimated. In addition, appearance of limbs highly varies due to changes in clothing and body shape (with the extreme and usual case of self occlusions), as well as changes in viewpoint manifested as 2D non-rigid deformations. Moreover, dynamically changing backgrounds of real-world scenes make data association complex among different frames. These difficulties have been addressed in several ways depending on the input data provided. Sometimes, 3D information is available because multiple cameras could be installed in the scene. Nowadays, a number of human pose estimation approaches from depth maps are also being published since the recent market release of low cost depth cameras [9]. In both cases, the problem is still challenging but ambiguities related to the 2D image projection are avoided since 3D data can be combined with RGB information. In many applications, however, only one camera is available. In such cases, either only RGB data is considered when still images are available, or it can be combined with temporal information when input images are provided in a video sequence.

The most of pose recovery approaches recover the human body pose in the image plane. However, recent works go a step further and they estimate the human pose in 3D [10]. Probably, the most challenging issue in 3D pose estimation is the projection ambiguity of 3D pose from 2D image evidences. This problem is particularly difficult for cluttered and realistic scenes with multiple people, were they appear partially or fully occluded during certain intervals of time. Monocular data is the less informative input to address the 3D pose recovery problem, and there is not a general solution for cluttered scenes. There exist different approaches, depending on the activity that people in the video sequence are carrying out. However, we found a lack of works taking into account the activity, the task or the behavior to refine the general approach.

Body pose recovery approaches can be classified, in a first step, between model based and model free methods. On the one hand, model free methods [11,12] are those which learn a mapping between appearance and body pose, leading to a fast performance and accurate results for certain actions (ex. walking poses). However, these methods are limited by background subtraction pre-processing or by poor generalization about poses that can be detected. On the other hand, most of the human pose estimation approaches can be classified as model based methods because they employ human knowledge to recover the body pose. Search space is reduced, for example, by taking into account the human body appearance and its structure, depending on the viewpoint, as well as on the human motion related to the activity which is being carried out.

**Figure 1.**
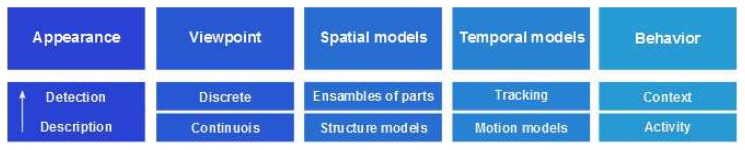
Proposed taxonomy for model-based Human Pose Recovery approaches.

In order to update recent advances in the field of human pose recovery, we provide a general and standard taxonomy to classify the State-of-the-Art of (SoA) model based approaches. The proposed taxonomy is composed of five main modules: appearance, viewpoint, spatial relations, temporal consistence, and behavior. Since this survey analyzes computer vision approaches for human pose recovery, image evidences should be interpreted and related to some previous knowledge of the body appearance. Depending on the appearance detected or due to spatio-temporal post processing, many works infer a coarse or a refined viewpoint of the body, as well as other pose estimation approaches restrict the possible viewpoints detected in the training dataset. Since the body pose recovery task implies the location of body parts in the image, spatial relations are taken into account. In the same way, when a video sequence is available, the motion of body parts is also studied to refine the body pose or to analyze the behavior being performed. Finally, the block of behavior refers, on the one hand, to those methods that take into account particular activities or the information about scene to provide a feedback to the previous modules, improving the final pose recognition. On the other hand, several works implicitly take into account the behavior by the election of datasets containing certain activities. The global taxonomy used in the rest of the paper is illustrated in Figure 1.

The rest of the paper is organized as follows: Section 2 reviews the SoA methods, categorized in the proposed taxonomy. In Section 3 we perform a methodological comparison of the most relevant works according to the taxonomy and discuss their advantages and drawbacks, and the main conclusions are found in Section 4.

## 2. State of the Art

Human pose recovery refers to the process of estimating the configuration of the body parts of a person (3D pose recovery) or their 2D projection onto the image plane (2D pose recovery). In general terms, Human Pose Recovery is the estimation of the skeleton which correctly fits with the image evidences. This process can be preceded by detection and tracking phases, typically used in pedestrian detection applications. Though an initial detection phase usually reduces the computation time of the system, it highly reduces the possible poses which can be estimated. For more information related to these topics refer to surveys on human detection and tracking [5,13,14].

Pose estimation surveys also exit in the literature [15–17], as well as more general studies involving recent works on vision-based human motion analysis [1,18]. All of them provide their own taxonomy. In [18], research is divided in two categories, 2D and 3D approaches, while [1] defines a taxonomy with three categories: model-free, indirect model use, and direct model use. As far as we know, work in [16] can be considered the most complete survey in the literature. They define taxonomies for model building (a likelihood function) and estimation (the most plausible pose given a likelihood function).

In the next subsections, the SoA related to human pose recovery is reviewed and model based works are classified according to the main modules proposed in [17]: *Appearance, Viewpoint, Spatial relations, Temporal relations* and *Behavior*. Furthermore, subgroups are defined for each taxonomy's module. See Figure 1.

### 2.1. Appearance

Appearance can be defined as image evidences related to human body and its possible poses. *Evidences* are not only referred to image features and input data, but also to pixel labels obtained from a certain labeling procedure. Hence, image evidences can be considered at different levels, from pixel to region and image. Description of image features and human (or body part) detections are both considered image evidences. The appearance of people in images varies among different human poses, lighting and clothing conditions, and changes in the point of view, among others. Since the main goal is the recovery of the kinematic configuration of a person, research described in this section tries to generalize over these kinds of variations.

**Figure 2.**
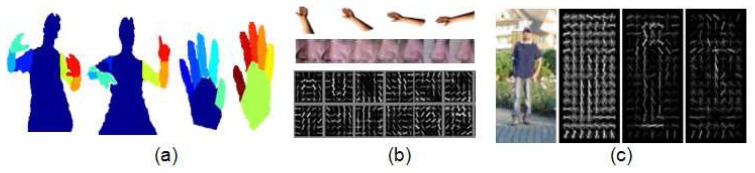
Examples of descriptors applied at pixel, local and global levels, respectively: (**a**) Graph cut approach for body and hands segmentation (frame extracted from [21]); (**b**) Steerable part basis (frame extracted from [25]); and (c) Image of a person and its HOG descriptor, and this descriptor weighted by the positive and negative classification areas (frame extracted from [26]).

Prior knowledge of pose and appearance is required in order to obtain an accurate detection and tracking of the human body. This information can be codified in two sequential stages: *description* of the image and *detection* of the human body (or parts), usually applying a previous learning process. The entire procedure from image description to the detection of certain regions can be performed at three different levels: pixel, local and global (shown in Figure 2a–c). Respectively, they lead to image segmentation [19–21], detection of body parts [22–25] and full body location [26,27]. It is widely accepted that describing the human body as an ensemble of parts improves the recognition of human body in complex poses, despite of an increasing of computational time. By contrast, global descriptors are successfully used in the human detection field, allowing fast detection of certain poses (e.g., pedestrians), as well as serving as initialization in human pose recovery approaches. The sub-taxonomies for both *description* and *detection* stages are detailed next.

#### 2.1.1. Description

Information extracted from images in the description phase will be analyzed in the detection stage. Typical methods applied for describing image cues are detailed below.

- **Silhouettes and contours** Silhouettes and their boundaries (edges and contours) provide powerful descriptors invariant to changes in color and texture. They are used to fit the human body in images [28] because most of the body pose information remains in its silhouette. However, these methods suffer from bad and noisy segmentations in real-world scenes, as well as the difficulty of recovering some Degrees of Freedom (DOF) because of the lack of depth information.
- **Intensity, color and texture** On one hand, gradients on image intensities are the most widely applied features for describing the appearance of a person. Histogram of Oriented Gradients (HOG) and SIFT descriptors use to be considered [26]. On the other hand, color and texture information by themselves can be used as additional cues for local description of regions of interest [10]. Color information is usually codified by means of histograms or space color models [29], while texture is described using Discrete Fourier Transform (DFT) [30] or wavelets such as Gabor filters [31], among others.
- **Depth** Recently, depth cues have been considered for human pose recognition since depth maps can be available from the multi-sensor Kinect™. This new depth representation offers near 3D information from a cheap sensor synchronized with RGB data. Based on this representation, new depth and multi-modal descriptors have been proposed, as well as classical methods has been revisited taking advantage of new visual cues. Examples are Gabor filters over depth maps for hand description [32] or novel keypoint detectors based on saliency of depth maps [33]. These approaches compute fast and discriminative descriptions by detecting extrema of geodesic maps and compute histograms of normal vectors distribution. However, they require an specific image cue, and depth maps are not always available.
- **Motion** Optical flow [34] is the most common feature used to model path motion and it can be used to classify human activities [35,36]. Additionally, other works track visual descriptors and codify the motion provided by certain visual regions as an additional local cue [37]. In this sense, following the same idea of HOG, Histogram of Optical Flow (HOF) can be constructed [35] to describe regions, as well as body parts movements.
- **Logical** It is important to notice that new descriptors including logical relations have been recently proposed. This is the case of the Group-lets approach by Yao and Fei-Fei [38], where local features are codified using logical operators, allowing an intuitive and discriminative description of image (or region) context.

#### 2.1.2. Detection

This stage refers to these specific image detections or output of classifiers which codify the human information in images. This synthesis process can be performed in four general areas summarized below.

- **Discriminative classifiers** A common technique used for detecting people in images consists of describing image regions using standard descriptors (*i.e.*, HOG) and training a discriminative classifier (e.g., Support Vector Machines) as a global descriptor of human body [26] or as a multi-part description and learning parts [39]. Some authors have extended this kind of approaches including spatial relations between object descriptors in a second level discriminative classifier, as in the case of *poselets* [27].
- **Generative classifiers** As in the case of discriminative classifiers, generative approaches have been proposed to address person detection. However, in the case of generative approaches they use to deal with the problem of person segmentation. For instance, the approach by Rother, Kolmogorov and Blake [40] learns a color model from an initial evidence of a person, as well as background objects, to optimize a probabilistic functional using Graph Cuts.
- **Templates** Example-based methods for human pose estimation have been proposed to compare the observed image with a database of samples [10].
- **Interest points** Salient points or parts in the images can also be used to compute the pose or the behavior is being carried out in a video sequence [37]. In this sense, we refer the reader to [41] for a fair list of region detectors.

### 2.2. Viewpoint

Viewpoint estimation is not only useful to determine the relative position and orientation among objects (or human body) and camera (*i.e.*, camera pose), but also to significantly reduce the ambiguities in 3D body pose [10]. Although in the literature the term *camera pose* is usually referred to as *pose*, we prefer to explicitly distinguish camera pose from pose as referred to human body posture, used throughout this review.

Usually, body viewpoint is not directly estimated in human tracking or pose recovery literature, however, it is indirectly considered. In many some, the possible viewpoints to be detected are constrained, for example, in the training dataset. Many woks can be found in upper body pose estimation and pedestrian detection literature, where only front or side views are respectively studied. As an example, the detector in [23] is presented as able to detect people in arbitrary views, however its performance is only evaluated on walking side views. Other works explicitly restrict their approaches to a reduced set of views, such as frontal and lateral viewpoints [42]. In those cases where the data set is composed of motion captures taken from different views without a clear discrimination among them, we consider that the viewpoint is neither explicitly nor implicitly considered.

Research where 3D viewpoint is estimated is divided in discrete classification and continuous viewpoint estimation (Figure 1).

#### 2.2.1. Discrete

**Figure 3.**
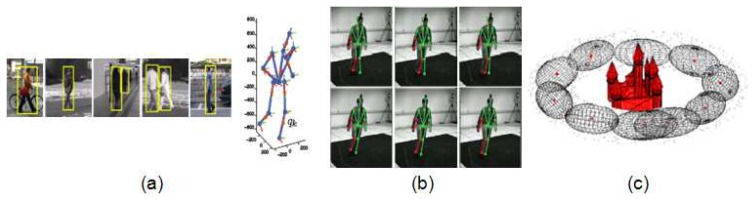
Viewpoint estimation examples: (**a**) First (discrete) and (**b**) second (continuous) phase of viewpoint estimation (frame extracted from [10]); and (**c**) Clusters of the camera pose space around the object which provide continuous viewpoint (frame extracted from [46]).

The discrete approach is treated as a problem of viewpoint classification category, where the viewpoint of a query image is classified into a limited set of possible initially known [43,44] or unknown [45] views. In these works, the 3D geometry and appearance of objects is captured by grouping local features into parts and learning their relations. Image evidences can also be used to directly categorize the viewpoint. In the first stage of the work by Andriluka, Roth and Schiele [10], a discrete viewpoint is estimated for pedestrians by training eight viewpoint-specific people detectors (shown in Figure 3a). In the next stage, this classification is used to refine the viewpoint in a continuous way (shown in Figure 3b), estimating the rotation angle of the person around the vertical axis by the projection of 3D exemplars onto 2D body parts detections.

#### 2.2.2. Continuous

The continuous approach to viewpoint estimation refers to estimating the real valued viewpoint angles for an example object or human in 3D.

Continuous viewpoint estimation is widely studied in the field of shape registration, which refers to finding correspondences between two sets of points and recovering the transformation that maps one point set to the other. Monocular non-rigid shape registration [47] can be seen as a similar problem to body pose estimation, since points in the deformable shape can be interpreted as body joints [48]. Given static images, the simultaneous continuous camera pose and shape estimation was studied for rigid surfaces [46], as well as for deformable shapes [49]. In both works, prior knowledge of the camera was provided by modeling the possible camera poses as a Gaussian Mixture Model (shown in Figure 3c).

### 2.3. Spatial Models

Spatial models encode the configuration of the human body in a hard (e.g., skeleton, bone lengths) or a soft way (e.g., pictorial structures, grammars). On one hand, structure models are mostly encoded as 3D skeletons and accurate kinematic chains. On the other hand, degenerative projections of the human body in the image plane are usually modeled by ensembles of parts. Independently of the chosen strategy, human pose recovery refers to the estimation of the full body structure, but also to the torso or upper body estimate. Since in TV shows and many scenes on films legs do not appear in the visible frame, several works [50,51] and datasets [52] have been restricted to upper body estimation.

#### 2.3.1. Ensembles of Parts

Techniques based on ensembles of parts consist of detecting likely locations of different body parts corresponding to consistent, plausible configuration of the human body. However, such composition is not defined by physical body constraints but rather by possible locations of the body parts in the image, so such techniques can deal with a high variability of body poses and viewpoints.

**Figure 4.**
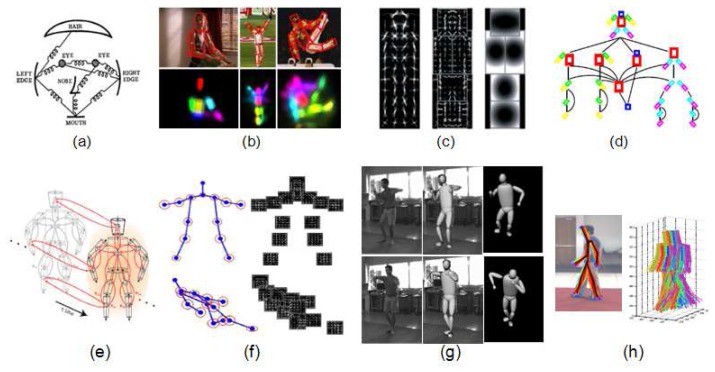
Examples of body models as a ensembles of parts: (**a**) Original (frame extracted from [53]) and (**b**) extended (frame extracted from [23]) Pictorial Structures; (**c**) Human model based on grammars: coarse filter (left), different part filters with higher resolution (middle), and model for spatial locations of parts (right) (frame extracted from [39]); (**d**) Hierarchical composition of body “pieces” (frame extracted from [24]); (**e**) Spatio-temporal loopy graph (frame extracted from [55]); (**f**) Different trees obtained from the mixture of parts (frame extracted from [56]); Structure models: (**g**) Two samples of 3D pose estimation during a dancing sequence (frame extracted from [57]); (**h**) Possible 3D poses (down) that match whose 2D projection (up) matches with detected body parts (frame extracted from [48]).

Pictorial structures [53] are generative 2D assemblies of parts, where each part is detected with its specific detector (shown in Figure 4a,b). Pictorial structures are a general framework for object detection widely used for people detection and human pose estimation [23,54]. Though the traditional structure for representation is a graph [53] (shown in Figure 4a), more recent approaches represent the underlying body model as a tree, due to inference facilities studied in [54]. Constraints between parts are modeled following Gaussian distributions, which do not seem to match, for example, with the typical walking movement between thigh and shank. However, Gaussian distribution does not correspond to a restriction in the 2D image plane: it is applied in a parametric space where each part is represented by its position, orientation and scale [54].

Grammar models as formalized in [58] provide a flexible and elegant framework for detecting objects [39], also applied for human detection in [39,59,60]. Compositional rules are used to represent objects as a combination of other objects. In this way, human body could be represented as a composition of trunk, limbs and face; as well composed by eyes, nose and mouth. From a theoretical point of view, deformation rules leads to hierarchical deformations, allowing the relative movement of parts at each level; however, deformation rules in [39] are treated as pictorial structures (shown in Figure 4c). Which makes grammars attractive is their structural variability while dealing with occlusions [59]. Following this compositional idea, [24] is based on *poselets* [27] to represent the body as a hierarchical combination of body “pieces” (shown in Figure 4d).

Ensembles of parts can also be performed in 3D when the 3D information is available from multi-camera systems [55,61]. An extension to pictorial structures in 3D is presented in [61], where temporal evolution is also taken into account (shown in Figure 4e). Joints are modelled following Mixture of Gaussian distributions, however it is named “loose-limbed” model because of the loosely attachment between limbs.

A powerful and relatively unexplored graphical representation for human 2D pose estimation are AND-OR graphs [62], which could be seen as a combination between Stochastic Context Free Grammar and multi-level Markov Random Fields. Moreover, their structure allows a rapid probabilistic inference with logical constrains [63]. Much research has been done in the graph inference area, optimizing algorithms to avoid local minima. Multi-view trees represent an alternative because a global optimum can be found using dynamic programming [56], hard pose priors [64] or branch and bound algorithms [65]. Moreover, in [56], parameters of the body model and appearance were learned simultaneously [56] in order to deal with high deformations of human body and changes in appearance (shown in Figure 4f).

#### 2.3.2. Structure Models

Due to the efficiency of trees and similarity between human body and acyclic graphs, most of the structure models are represented as kinematic chains following a tree configuration. Contrarily to the trees explained above, whose nodes represent body parts, nodes of structure trees usually represent joints, each one parameterized with its degrees of freedom (DOF). In the same way that ensembles of parts are more frequently considered in 2D, accurate kinematic constraints of structure models are more appropriate in a 3D representation. However, the use of 2D structure models is reasonably useful for motions parallel to the image plane (e.g., gait analysis [42]). 2D pose is estimated in [66] with a degenerate 2D model learned from image projections.

3D recovery of human pose from monocular images is the most challenging situation in human pose estimation due to projection ambiguities. Since information is lost during the projection from real world to the image plane, several 3D poses match with 2D image evidences [57]. Kinematic constraints on pose and movement are typically employed to solve the inherent ambiguity in monocular human pose reconstruction. Therefore, different works have focused on reconstructing the 3D pose given the 2D joint projections from inverse kinematics [67,68], as well as the subsequent tracking [69,70]. In [69], the human body is modelled as a kinematic chain, parameterized with twists and exponential maps. Tracking is performed in 2D, from a manual initialization, projecting the 3D model into the image plane under orthographic projection. This kinematic model is also used in [71], adding a refinement with the shape of garment, providing a fully automatic initialization and tracking. However this multi-camera system requires a 3D laser range model of the subject which is being tracked. In [57], 3D pose is estimated by projecting a 3D model onto the image plane in the most suitable view, through perspective image projection (shown in Figure 4g). The computed kinematic model is based on hard constraints on angle limits and weak priors, such as penalties proportions and self collisions, inspired in a strong human knowledge.

The recovered number of Degrees of Freedom (DOF) varies greatly among different works, from 10 DOF for upper body pose estimation, to full-body with more than 50 DOF. However, the number of possible poses is huge even for a model with few DOF and a discrete parameter space. Because of this reason, kinematic constraints such as joint angle limits are typically applied over structure models. Other solutions rely on reducing the dimensionality applying unsupervised techniques as Principal Component Analysis (PCA) over the possible 3D poses [42,48,66,72]. The continuous state space is clustered in [66], and PCA is applied over each cluster in order to deal with non-linearities of the human body performing different actions. As well as in [42], where it is used a Hierarchical PCA depending on human pose, modeling the whole body as well as body parts separately.

Hybrid approaches also exist, which exploit the benefits of both structure models and ensembles of parts (shown in Figure 4h). Following the ideas of shape registration field, structural models in [48] are learned from body deformations of different human poses, followed by a PCA in order to reduce the dimensionality of the model. Moreover, the search space of possible poses is reduced by taking profit of SoA body part detectors proposed in [56].

With the same intention, parameters of the structural model and appearance can be learned simultaneously. Active Shape Models (ASM) [73] and Active Appearance Models (AAM) [74] are labelled models which are able to deform their shape according to statistical parameters learned from the training set. AAM, moreover, are able to learn the appearance surrounding the anatomical landmarks, reliably labelled in the training examples. Though ASM and AAM are mostly used for face detection and head pose estimation [75], the learning of local appearance and deformations of body parts is also used for body pose estimation [76]. These approaches use to provide a higher degree of generalization than example-based approaches, which compare the image evidences with a database of samples. While the body parts detection in [10] is performed by multi-view pictorial structures, 3D reconstruction is estimated by projecting 3D examples over the 2D image evidence.

### 2.4. Temporal Models

In order to reduce the search space, temporal consistence is studied when a video sequence is available. Motion of body parts may be incorporated to refine the body pose or to analyze the behavior that is being performed.

#### 2.4.1. Tracking

Tracking is applied to ensure the coherence among poses over the time. Tracking can be applied separately to all body parts or only a representative position for the whole body can be taken in account. Moreover, 2D tracking can be applied to pixel or world positions, *i.e.*, the latest when considered that the person is moving in 3D. Another subdivision of tracking is the number of hypothesis, which can be a single one maintained over the sequence or multiple hypothesis propagated in time.

**Figure 5.**
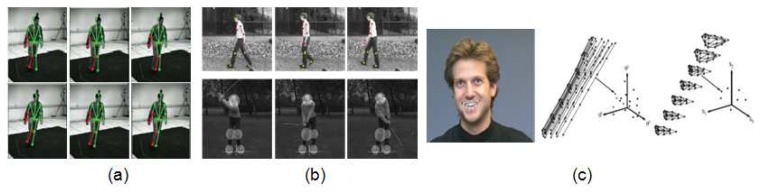
Examples of tracking sequences: (**a**) 3D tracking of the whole body, through a multiple hypothesis approach (frame extracted from [10]); (**b**) 2D tracking of body parts (frame extracted from [77]); (**c**) left: 3D features on a smiling mouth; right: a comparison of shape and trajectory space (frames extracted from [78]).

Single tracking is applied in [42], where only the central part of the body is estimated through a Hidden Markov Model (HMM). Finally the 2D body pose is recovered from the refined position of the body. Also in 2D, a single hypothesis by each body joint (shown in Figure 5b) is propagated in [77]. Though both approaches are performed in 2D, they do not loose generality at these stage since they work with movements parallel to the image plane. In contrast, 3D tracking with multiple hypotheses is computed in [10], leading to a more accurate and consistent 3D body pose estimation (shown in Figure 5a).

In the topic of shape recovery, a probabilistic formulation is presented in [79] which simultaneously solves the camera pose and the non-rigid shape of a mesh (*i.e.*, body pose in this topic) in batch. Possible positions of landmarks (*i.e.*, body parts) and their covariances are propagated along all the sequence, optimizing the simultaneous 3D tracking for all the points.

#### 2.4.2. Motion Models

The human body can perform a huge diversity of movements, however, specific actions could be defined by smaller sets of movements (e.g., in cyclic actions as walking). In this way, a set of motion priors can describe the whole body movements when a single action is performed. However, hard restrictions on the possible motions recovered are as well established [66,72].

Motion models are introduced in [80], combined with body models of walking and running sequences. A reduction of dimensionality is performed by applying PCA over sequences of joint angles from different examples, obtaining an accurate tracking. This work is extended in [81] for golf swings from monocular images in a semi-automatic framework. Scaled Gaussian Process Latent Variable Models (SGPLVM) can also represent more different human motions [82] for cyclic (ex. walking) and acyclic (ex. golf swing) actions, from monocular image sequences, despite of imposing hard priors on pose and motion. In [83], for instance, the problem of pose estimation has been addressed from the temporal domain. Possible human movements have been learned through a Gaussian Process, reducing the search space for pose recovery while performing activities such skiing, golfing or skating.

A potential issue of motion priors is that the variety of movements that can be described highly depends on the diversity of movements in the training data. On the other hand, a general trajectory based on the Discrete Cosine Transform (DCT) is introduced in [84] to reconstruct different movements from, for example, faces and toys (shown in Figure 5c). In this case, trajectory model is combined with spatial models of the tracked objects. Applications of such motion models related to human pose can be found in [85], where it is achieved a 3D reconstruction of moving points tracked from humans and scenes; as well in [86], where articulated trajectories are reconstructed for upper body models.

### 2.5. Behavior

The block of behavior in our taxonomy refers to those methods that take into account activity or context information to provide a feedback to previous pose recognition modules [87]. Most approaches previously described do not directly include this kind of information. However, databases are usually organized by actions (e.g., walking, jogging, boxing [88]) and algorithms use to learn poses belonging to some of these actions (e.g., walking [10], golf swings [81]). In this sense, the selection of a specific training dataset is a direct or indirect choice of the set of actions that the system will be able to detect. It is important to point out that taxonomies in the literature for behavior, action, activity, gesture and sub-gesture, for example, are not broadly detailed. The term *behavior* is used here as a general concept which includes actions and gestures.

Though behavior analysis is not usual in the SoA of pose estimation, some works take into account behavior or activity to estimate an accurate body pose, learning different models depending on the action that is being performed [72]. Different subspaces are computed for each action in [66]. However, the number of actions chosen is a critical parameter, since actions seen from different viewpoints are interpreted as different movements. This phenomenon occurs because a degenerate 2D model is learned from image projections, instead of building a 3D view invariant model.

**Figure 6.**
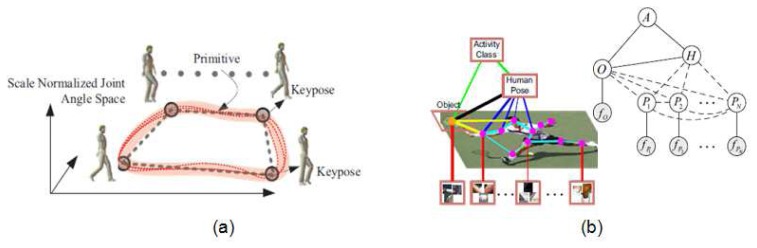
Joint human pose and behavior estimation: (**a**) Different walking examples (curves), the learned models (piecewise lines) and its key poses (frame extracted from [6]); (**b**) Graphical model proposed for object detection (O) and human pose estimation (H) from body part (*P_i_*) detections, and an image example of a human playing tennis (frame extracted from [89]).

Some works in the literature go a step forward and jointly recover pose and behavior. In the work by Yao and Li [89], the authors include context information about human activity and its interaction with objects (shown in Figure 6b) to improve both the final pose estimation and activity recognition. They report that ambiguities among classes are better discriminated. Similarly, Andriluka and Sigal extended in [90] their previous work in multi-people 3D pose estimation by modelling the human interaction context. They achieved successful results on competition and dancing videos by treating detected subjects as mutual “context” for the other subjects in the scene.

Finally, the work by Singh and Nevatia [6] takes profit from a joint estimation of the human pose and the action being performed. A set of key poses are learned for each action (shown in Figure 6a) and the 3D pose is accurately recovered using the specific model for such action, showing how joint estimation of behavior and pose can improve both results.

## 3. Discussion

**Figure 7.**
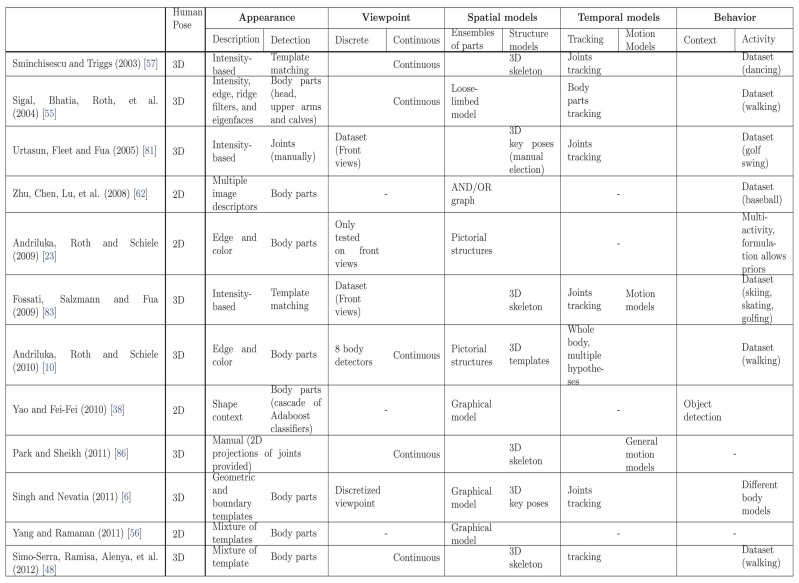
Comparative of model based Human Pose Recovery approaches. Dashes in *Viewpoint* and *Behavior* indicate that the corresponding work does not study the module described in the column. Dashes in *Temporal models* mean that the video sequence is not available in the corresponding work.

Human pose recovery is a challenging problem because of the many degrees of freedom to be estimated, changes in appearance and viewpoint, and the huge number of poses and movements that humans can perform. In order to review the current trends in this field, the most relevant works are compared in Figure 7.

All the listed methods can not be compared based on their performance results because it does not exist a common benchmark to compare 2D and 3D pose estimation approaches, as well as the joint estimation of human pose and behavior. Moreover, some of them have the best current results, while other works, which have been overcame by more recent techniques, have been significant to advance the SoA. Hence, the comparison presented in Figure 7 tackles their methodologies, according tho the taxonomy proposed in Figure 1.

Work in [10] is an example of using models with excellent results. They modeled almost each module of our proposed taxonomy, outperforming the SoA. Their approach rely on using strong body part detectors in conjunction with a powerful 3D tracking.

By contrast, in [48] a 3D pose estimation approach from still images is proposed. They report good estimates of the human pose in video frames where [10] fails. They used similar body part detectors but, instead of modeling human dynamics, they modeled the possible body deformations, penalizing non-anthropomorphic poses.

In the case of 2D pose estimation, the best results in the SoA are achieved in [56]. A fast approach based on strong body part detectors and a flexible tree configuration is proposed, encoding pairwise relations between consecutive body parts. Following with still images, excellent results are achieved in [89] by using behavior or context information through object detection. However, the same image descriptor is used for objects and body parts, and the current SoA of image descriptors oriented to body parts [56] could be used to improve their results.

From a global point of view, the performance of model based approaches for human pose recovery rely specially on the Appearance module, *i.e.*, image description and body part detectors. However, though the SoA body part detectors have reported impressing results, they find many false positives. Hence, the goal of spatial models is the restriction of such image evidences to find the specific combination that composes the human body. On the one hand, best performance's 2D pose estimators model the human body as an ensemble of parts. On the other hand, works computing 3D pose require 3D structure models limited by physical or anthropomorphic constraints. At this point, approaches with less sophisticated spatial models also impose temporal or viewpoint constraints to reduce the search space.

In order to complete this survey, a discussion referred to current trends for each one of the taxonomy's modules is detailed below.

- **Appearance** It is widely accepted that the best current results for this module are achieved by body part detectors. However, there is not consensus on the best descriptor. Though tracking-based approaches tends to use simple descriptors based on intensity, the most of current works consider HOG and derivative-based approaches to describe local image evidences. In [56] HOG statistics are considered, so flexible body part detectors are built through combinations of HOG basis filters, which can deal with high variability of human appearance.
- **Viewpoint** Most referred methods for viewpoint analysis have been split into discrete and continuous techniques. Viewpoint is commonly estimated in 3D human pose recovery approaches and it is not as usual in works where body pose is computed in 2D. Moreover, the huge variability of 3D human poses makes their projection to the 2D image plane highly nonlinear [48]. Simultaneous 3D human pose recovery and camera pose estimation [49] is an elegant approach to reduce such nonlinearities.
- **Spatial models** Spatial models were reviewed and divided into ensembles of parts and structure models, depending on their flexibility. Ensemble of parts approaches result very useful to fit with 2D image evidences, since they occur in a 2D degenerative space where accurate kinematic constraints are too hard to deal with the huge amount of body movements, combined with changes in viewpoint and projection. Structure approaches can deal with 3D pose more accurately, reducing the search space through physical constraints. To this end, current parametric 3D skeletons [48] and key poses [10] show similar results. In the past years, pictorial structures predominated in the SoA. However, recent approaches based on multi-view trees [56] and grammars [39] provide interesting frameworks to deal with occlusions, high variability of human poses and the large amount of false positives provided by body part detectors.
- **Temporal models** Temporal models were reviewed and split them into tracking and motion models. When video sequences are available, 3D information in tracking approaches improves 2D methods since nonlinearities due to viewpoint projection are reduced. Hard motion priors help in the pose estimation problem, reducing the search space despite of limiting the possible movements that can be detected.
  General motion models help also to reduce the search space of body configurations. However, they were not tested under the same conditions (Figure 7). The appearance module is avoided since key points in images were previously provided [86]. In this way, general models deserve further study about their application with body part detectors and noisy input data.
- **Behavior** The common approach to include human behavior in pose estimation methods is by constraining datasets to certain activities. However, neither simultaneous estimation of behavior and human pose, nor the human pose refinement activity estimation are common in the literature. Scene understanding has recently demonstrated to be a powerful field of research which provides a useful feedback between the problem of object recognition and the human pose recovery problem [89]. This kind of inference can be incorporated in a higher layer of knowledge (*i.e.*, an “ambient intelligence” layer) where context, scene and activity information can provide valuable feedback to any module of the approach to improve the final pose estimation process.

## 4. Conclusions

In this survey, we have reviewed past and current trends in the field of human pose recovery. We have proposed a new taxonomy and grouped SoA model based methods into appearance, viewpoint, spatial relations, temporal consistence, and behavior modules. Moreover, a methodological comparison was performed following the proposed taxonomy, evaluating current SoA approaches in the aforementioned five group categories.

*Appearance* is the most stable area because of the widely extended use of edge based descriptors (e.g., HOG) to detect body parts. By contrast, current trends for *spatial models* are diverse. Very different representations of spatial relations among body parts are combined with a high variety of inference methods, drawing a heterogeneous SoA. *Temporal models* module is clearly oriented to tracking, which is predominant to motion model approaches when video sequences are available. Indeed, motion models have not been deeply explored in the field of human pose estimation, though they could be used to reduce the huge search space of some approaches for 3D human pose recovery.

Both *viewpoint* and *behavior* modules are less present in the literature. However, since a joint viewpoint and 3D pose estimation is a hard problem, it could be used to reduce the nonlinearities of the estimation problem. In the same way, a joint behavior and body pose analysis is becoming a common trend to improve the generalization capability of current approaches, thus including the context as a complementary and discriminative source of information. In other words, future trends in human pose recovery will tend to combine the knowledge of the global scene and objects nearby together with the detected human pose and their analyzed motion.
